# Circulating hepassocin level in patients with stable angina is associated with fatty liver and renal function

**DOI:** 10.7150/ijms.50646

**Published:** 2021-01-01

**Authors:** I-Ting Tsai, Wei-Chin Hung, Yung-Chuan Lu, Cheng-Ching Wu, Thung-Lip Lee, Chin-Feng Hsuan, Teng-Hung Yu, Ching-Ting Wei, Fu-Mei Chung, Yau-Jiunn Lee, Chao-Ping Wang

**Affiliations:** 1 Department of Emergency, E-Da Hospital, Kaohsiung 82445 Taiwan.; 2 Division of Cardiology, Department of Internal Medicine, E-Da Hospital, Kaohsiung 82445 Taiwan.; 3 Division of Endocrinology and Metabolism, Department of Internal Medicine, E-Da Hospital, Kaohsiung 82445 Taiwan.; 4 Division of General Surgery, Department of Surgery, E-Da Hospital, Kaohsiung 82445 Taiwan.; 5 School of Medicine, College of Medicine, I-Shou University, Kaohsiung, 82445 Taiwan.; 6 The School of Chinese Medicine for Post Baccalaureate, College of Medicine, I-Shou University, Kaohsiung, 82445 Taiwan.; 7 School of Medicine for International Students, College of Medicine, I-Shou University, Kaohsiung, 82445 Taiwan.; 8 Department of Biomedical Engineering, I-Shou University, Kaohsiung, 82445 Taiwan.; 9 Department of Electrical Engineering, I-Shou University, Kaohsiung, 82445 Taiwan.; 10 Lee's Endocrinologic Clinic, Pingtung 90000 Taiwan.; 11 Division of Cardiology, Department of Internal Medicine, E-Da Cancer Hospital, Kaohsiung 82445 Taiwan.

**Keywords:** Hepassocin, fatty liver, renal function, stable angina

## Abstract

**Background:** Chronic kidney disease (CKD) is a major risk factor for coronary artery disease and it is often associated with hepatic steatosis. Hepassocin (also known as hepatocyte-derived fibrinogen related protein or fibrinogen-like 1) is a novel hepatokine that causes hepatic steatosis and induces insulin resistance. However, the role of hepassocin in renal function status remains unclear. Our objective was to investigate the association of plasma hepassocin level with fatty liver and renal function status in patients with stable angina.

**Methods:** Plasma hepassocin levels were determined by enzyme-linked immunosorbent assays in 395 consecutive patients with stable angina. Renal function was defined as an estimated glomerular filtration rate (eGFR). Fatty liver was defined by ultrasonography and fibrosis-4 (FIB-4) index.

**Results:** With increasing hepassocin tertiles, patients had higher prevalence of fatty live, an increased waist-to-hip ratio, and neutrophil count, monocyte count, and FIB-4 index, higher levels of uric acid, blood urine nitrogen and higher sensitivity C-reactive protein. They also had incrementally lower eGFR, serum hemoglobin and albumin levels. In multiple linear stepwise regression analysis, only eGFR was significantly independent negatively associated with plasma hepassocin levels.

**Conclusion:** Our results indicate that circulating hepassocin in patients with stable angina is associated with fatty liver and renal function, which suggests that increased plasma hepassocin may be involved in the pathogenesis of fatty liver and CKD.

## Introduction

Chronic kidney disease (CKD) is an independent risk factor for coronary artery disease 1. There is a graded-independent association between reduced estimated glomerular filtration rate (eGFR) and the risk of death, cardiovascular events, and hospitalization 2. CKD has been a global public health issue for the past few decades and it affects more than 10% of the worldwide population. The health burden of CKD is not restricted to the need for renal replacement therapy for end-stage renal disease (ESRD), but also its other serious outcomes, such as cardiovascular events and death, which are strongly influenced by kidney involvement 3,4. Previous study reported that in 2010, death caused by CKD almost doubled compared with 1990 and it was ranked as the 18th highest risk factor for mortality 5. Taiwan has the highest incidence of ESRD, which requires renal replacement therapy, and CKD contributes to this burden. CKD patients have a chronic, low grade, inflammatory condition that is mediated by inflammatory cytokines 6. In the liver, hepatic inflammation triggers a cascade of hepatocyte injuries, ranging from simple hepatic steatosis to nonalcoholic steatohepatitis, cirrhosis and even hepatocellular carcinoma 7,8. In addition, subclinical chronic inflammation seems to be an independent risk factor for the development of stable angina in CKD patients 9. Although it is well known that CKD, nonalcoholic fatty liver disease (NAFLD) and cardiovascular disease are strongly connected, the factors linking these diseases are not yet fully understood.

Hepassocin, also known as hepatocyte-derived fibrinogen related protein or fibrinogen-like 1, is a specific mitogenic factor for hepatocytes. It has also been identified as a novel hepatokine, which increases hepatic lipogenesis that causes hepatic steatosis 10. In addition, hepassocin was demonstrated to induce insulin resistance (IR) in both the liver 11 and skeletal muscles 12. Although a previous study indicated that hepassocin is also expressed by adipose tissues, the association between hepassocin and renal function status remains obscure 13.

In view of the fact that hepassocin causes hepatic steatosis and induces IR, and because CKD has a clear association with both hepatic steatosis and type 2 diabetes 14,15, the authors postulated that hepassocin might be the link between these diseases. The aim of the present study was to clarify the clinical significance of circulating hepassocin levels in the context of fatty liver and renal function status. Circulating hepassocin levels were determined in patients with stable angina, and then the association between those levels and the fatty liver and renal function status were evaluated.

## Methods

### Study participants

A total of 414 consecutive patients with stable angina who were admitted to the cardiovascular ward of E-Da Hospital between June 2007 and June 2017 were prospectively enrolled in the study. The eGFRs were calculated using the CKD-EPI two-concentration race equation 16, and their renal function status was confirmed by follow-up eGFR measurements at 3 months after hospital discharge. Patients with a history of concomitant inflammatory disease (including malignancy, liver disease, sepsis, infection and collagen disease), steroid use or surgery within 1 month prior to admission, as determined by interviews, physical examinations, biochemical laboratory analysis and urinalysis, were excluded from the study. Furthermore, patients who were unable or unwilling to give informed consent were also excluded. After the exclusion of 19 patients, a total of 395 patients with stable angina were included in the present study. In addition, in the present study, no patients have undergone dialysis. Hypertension was defined as a resting systolic blood pressure of ≥140 mmHg, a diastolic blood pressure of ≥90 mmHg or both, and those with a history of hypertension and the use of anti-hypertensive drugs. Diabetic patients were defined as those who were currently being treated for diabetes, those with a fasting plasma glucose level ≥126 mg/dl on 3 separate days, or a casual glucose level ≥200 mg/dl. Hyperlipidemia was defined as a total cholesterol level of >200 mg/dl, and/or a low-density lipoprotein cholesterol (LDL-C) level of >130 mg/dl, and/or a high-density lipoprotein cholesterol (HDL-C) level of <40 mg/dl, and/or a triglycerides level of >180 mg/dl, or those undergoing treatment for lipid disorders. The study protocol was approved by the Human Research Ethics Committee at E-Da Hospital and all patients provided written informed consent before enrollment.

In the present study, all of the study patients were of Han Chinese origin and lived in the same region. All patients underwent complete routine biochemical analyses of their blood and urine, as well as physical examinations. Anthropometric parameters were taken, including their waist-to-hip ratio and their body mass index (BMI). Waist and hip circumferences were measured at the end of a normal respiration and each measurement was repeated twice. The measurements were recorded to the nearest 0.1 cm and the waist was measured at the narrowest point between the lower border of the ribs and the right iliac crest. Hip circumferences were measured at the widest point. Seated blood pressure was also measured by a trained nurse with a digital automatic blood pressure monitor (Omron model HEM-907, Omron, Japan) after the patients had rested for 5 minutes. Plasma biochemical parameters were measured after overnight fasting. Plasma total cholesterol, LDL-C, HDL-C, triglycerides, creatinine, glucose, and uric acid were measured using standard commercial methods as described in our previous reports 17,18. Patients who had smoked within one year of the examination were defined as current smokers and those who had stopped smoking for more than one year before the examination were defined as nonsmokers.

### Plasma hs-CRP and hepassocin measurements

All patient blood samples were drawn after overnight fasting, and all plasma samples were kept at -80 °C prior to subsequent assays. Plasma hepassocin concentration was measured using a commercial enzyme-linked immunosorbent assay (ELISA) kit (intra-assay CV <10%, inter-assay CV <12%; Cloud-Clone Corp., Katy, USA) and the ELISA was performed according to the manufacturer's instructions. According to the manufacturer, the hepassocin ELISA has excellent specificity for the detection of human hepassocin, and no significant interference with analogues or cross-reactivity was observed. In addition, plasma high sensitivity C-reactive protein (hs-CRP) was determined using a Beckman Coulter IMMAGE Immunochemistry System (Brea, CA, USA). Samples were measured in duplicate in a single experiment and the detection limit of the hs-CRP assay system was 0.2 mg/L.

### Liver ultrasonography and fibrosis-4 (FIB-4) index calculation

All participants underwent hepatic ultrasonography, which was performed by two experienced physicians who were blinded to the study aims. Hepatic steatosis was diagnosed according to the following sonographic features: evidence of diffuse hyperechogenicity in the liver relative to the kidneys, poor visualization of intrahepatic structures, and ultrasound beam attenuation. The FIB-4 index was calculated as follows: FIB-4 = age (years) x AST (IU/L) / platelet count (10^9^/L)/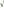
ALT (IU/L), as previously reported by Vallet-Pichard *et al.* 19.

### Statistical analysis

Data normality was analyzed using the Kolmogorov-Smirnov test. Continuous, normally distributed variables are described as the mean ± standard deviation. Non-normally distributed variables are described as the median (interquartile range). Statistical differences between normally distributed variables were compared using one-way analysis of variance (ANOVA) followed by Tukey pairwise comparison. Categorical variables are presented as frequencies and percentages, and inter-group comparisons were tested using the chi-square test. Logarithmically transformed values of plasma hepassocin, hs-CRP, blood urine nitrogen (BUN), creatinine, and triglyceride were used in the statistical analyses since their distributions were skewed. Associations between hepassocin and other parameters were assessed using simple and multiple linear stepwise regression analyses. Results were considered statistically significant if their p value was <0.05. All analyses were performed using SAS statistical software, version 8.2 (SAS Institute Inc., Cary, NC, USA).

## Results

### Main characteristics according to tertiles of hepassocin

**Table 1** shows the clinical characteristics of the 395 patients with stable angina (males, 69.6%; females, 30.4%) stratified by hepassocin. The mean hepassocin level was 1212.4 ng/mL and the median plasma hepassocin level was 903.0 ng/mL (interquartile range, 431.6-1413.9 ng/mL). The patients were divided according to the tertiles of hepassocin as follows: low hepassocin (≤570 ng/mL), n = 130; medium hepassocin (571-1230 ng/mL), n = 132; and high hepassocin (>1230 ng/mL), n = 133. The patients in the high hepassocin group were associated with higher prevalence of fatty liver and lower prevalence of single-vessel disease. Furthermore, the high hepassocin group had a higher waist-to-hip ratio compared with the low hepassocin group. There were no significant differences in sex, age, hypertension, diabetes mellitus, hyperlipidemia, current smoker, BMI, SBP, DBP, left ventricular ejection fraction, medications, two-vessel disease, and three-vessel disease among the three groups.

### Biochemical characteristics according to tertiles of hepassocin

The data presented in **Table 2** shows that as the hepassocin tertile increased, there were significant decreases in eGFR, hemoglobin and albumin concentrations, and significant increases in uric acid, BUN, and hs-CRP concentrations, as well as neutrophil and monocyte count and FIB-4 index. There were no significant differences in fasting glucose, HbA1C, triglyceride, HDL-cholesterol, LDL-cholesterol, creatinine, total WBC count, and lymphocyte count among the three groups.

### Association between plasma hepassocin levels and clinical laboratory data

**Figure 1:**
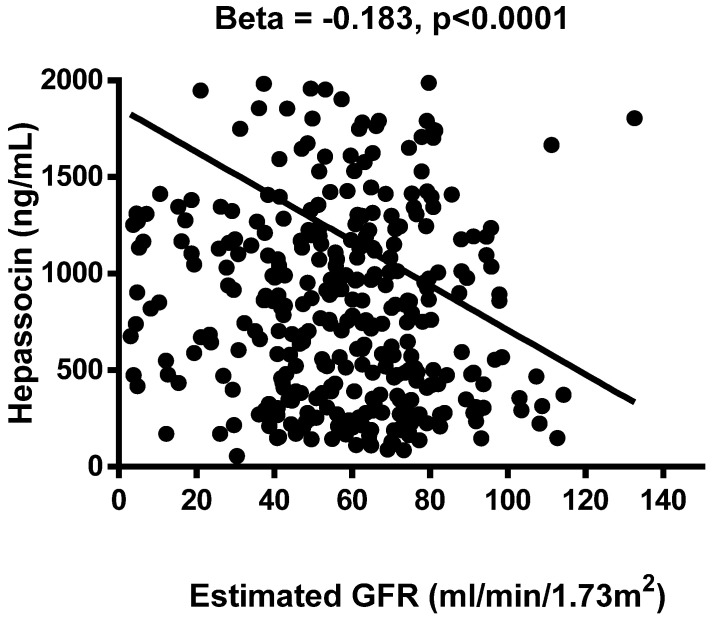
Association between plasma hepassocin concentration and estimated glomerular filtration rate (eGFR), plasma hepassocin concentration had a significant and negatively association with eGFR.

The results of the association analysis for circulating hepassocin and the relevant parameters are shown in **Table 3.** Plasma hepassocin was significantly positively related to uric acid (β = 0.137, *p =* 0.010), BUN (β = 0.139, *p =* 0.006), creatinine (β = 0.103, *p =* 0.040), total WBC count (β = 0.111, *p =* 0.028), neutrophil count (β = 0.106, *p =* 0.036), hs-CRP (β = 0.108, *p =* 0.041), and FIB-4 index (β = 0.122, *p =* 0.012), and significantly negatively related to eGFR (β = -0.183, *p <*0.0001, **Figure 1**) in the simple linear regression analysis with pooled data, whereas in the multiple linear stepwise regression analysis, only the plasma hepassocin level remained negatively associated with eGFR (β = -0.228, *p <*0.0001).

## Discussion

The present study demonstrated for the first time that plasma hepassocin levels associated with renal function status in patients with stable angina. Simple linear regression analysis revealed that hepassocin was positively associated with uric acid, BUN, creatinine, total WBC count, neutrophil count, hs-CRP, and FIB-4 index, and significantly negatively related to eGFR. Furthermore, in multiple linear stepwise regression analysis, only the plasma hepassocin level remained negatively associated with eGFR. With increasing hepassocin levels, the patients had higher rates of fatty liver, an increased waist-to-hip ratio, neutrophil count and monocyte count, and higher levels of uric acid and BUN as well as hs-CRP and FIB-4 index. With increasing hepassocin levels, patients also had incrementally lower eGFR, serum hemoglobin and albumin levels.

In the current study, the associations between plasma hepassocin levels and other relevant risk factors for renal function were evaluated. Hepassocin is a hepatokine 20 that plays an important role in the regulation of hepatocyte proliferation 21, and its expression is increased during liver regeneration 22. Previous study showed that in the regenerating liver may induce an acute-phase response and increased acute-phase cytokines 23. These cytokines may contribute to CKD. In addition, a previous study provided evidence that hepassocin plays an important role in NAFLD and induces hepatic lipid accumulation through an extracellular signal-regulated kinase 1/2 (ERK1/2)-dependent signaling pathway 24. Lu *et al.* reported that serum hepassocin concentrations are gradually increased in diabetic patients with NAFLD, and suggested that increased hepassocin levels might have clinical implications and could play a role as a biomarker for diabetes and NAFLD 25. Our recent study showed that a bidirectional relationship existed between CKD and NAFLD in patients with type 2 diabetes mellitus 14. Consistent with these findings, it was found in the present study that patients in the high hepassocin group were associated with increased rates of fatty liver and decreased eGFR.

Hepassocin is also expressed by brown adipose tissues, and signals generated following liver injury can also enhance hepassocin expression by brown adipose tissues, which suggests that there is cross talk between the injured liver and adipose tissues 26. In addition, it is known that interleukin-6 (IL-6) increases hepassocin expression 27, indicating that elevated hepassocin might also be involved in obesity-induced IR. Furthermore, Cheng *et al.* found that activation of signal transducer and activator of transcription-3 by IL-6 were mediated by hepassocin expression 28. In the current study, it was revealed that the high hepassocin group had a higher waist-to-hip ratio compared with the low hepassocin group, and with increasing hepassocin tertiles, there were significant increases in hs-CRP concentrations as well as neutrophil and monocyte counts. The results of the present study support the idea 29-31 that hepassocin may act through the IR and inflammation response to play an important role in the impact of renal function status in patients with stable angina.

To the best of our knowledge, this is the first report to demonstrate that hepassocin levels are associated with renal function status. The biological mechanisms by which hepassocin is involved in the impact of renal function are not well understood. The current study found an inverse correlation between plasma hepassocin levels and eGFR, however, whether unchanged hepassocin levels reflect impaired hepassocin clearance by the kidneys or a compensatory mechanism aimed at counteracting increased cardiovascular risk factors, is not clear. Previous studies have reported that hepassocin regulates cell fate and has a protective effect on CCl4-induced liver injury 32. In addition to hepassocin's role in the development of NAFLD, the hepassocin promoter is transcriptionally upregulated through an IL-6/IL-6R/STAT3 signaling pathway, which maintains metabolic homeostasis 33. In addition, NAFLD is strongly associated with obesity, diabetes and IR, and is considered to be the hepatic manifestation of metabolic syndrome 34,35. Hepassocin may therefore play a role in the regulation of insulin sensitivity or metabolic homeostasis. On the basis of these reports, patients with stable angina with elevated hepassocin levels had higher prevalence of fatty live and reduced renal function observed in the current study, could indicate that hepassocin may be associated with pathogenesis of fatty live and CKD.

There were several limitations to the current study. First, the study was cross-sectional in design, and it was not possible to clearly establish a causal link between plasma hepassocin levels and the development of CKD. Second, it is well known that the use of lipid-lowering agents and anti-diabetic medicines influences CKD. The association between patients receiving prescription medications among the tertiles of hepassocin was analyzed. There were no significant differences in angiotensin-converting enzyme inhibitors/angiotensin receptor blockers, calcium channel blocker, diuretics, thiazolidinediones, and statins treatments among the three groups. Hence, the possible influence of these medications on renal function cannot be ignored. Third, the current study included only patients with stable angina, and therefore the results may not be fully applicable to the general population. Fourth, the hepassocin molecular weight is 36 kDa that is belong to low molecular weight protein. Low molecular weight proteins may possibly eliminated by the kidney. However, previous studies demonstrated that low molecular weight human serum proteins, peptides, and other small components have been associated with pathological conditions such as cancer 36, diabetes 37, and cardiovascular and infectious diseases 38, and kidney disease 39. Hence, according to the results of the present study, hepassocin may play a role in the impact of renal function. But, further studies are needed to clarify the exact role of hepassocin in renal function status. Fifth, hepassocin expression is increased during liver regeneration and so hepassocin may be as an acute phase protein. Further work is required to confirm the other proteins including fibrinogen that could be of some help in clarifying the role of hepassocin as an acute phase protein and impact of renal function status. Finally, the analyses are based on single measurements of plasma hepassocin, which may not fully reflect the association over time. It would be interesting to measure serial changes in plasma hepassocin levels in CKD, obese or IR subjects to further clarify the role of hepassocin in the pathogenesis of CKD. Additional studies that include a larger multi-ethnic cohort are needed to investigate these associations.

## Conclusions

In conclusion, our findings of elevated hepassocin levels were associated fatty liver and renal function status, indicate that hepassocin may contributed to the pathogenesis of fatty liver and CKD in patients with stable angina. However, further studies are needed to clarify the exact role of hepassocin.

## Figures and Tables

**Table 1:** Main characteristics according to tertiles of hepassocin

Parameter	Low hepassocin ≤ 570 ng/mL	Medium hepassocin = 571-1230 ng/mL	High hepassocin >1230 ng/mL	*p*-value
No.	130	132	133	
Sex (male/female)	81/49	99/33	95/38	0.071
Age (years)	69.0±11.5	69.8±10.4	72.2±11.5	0.054
Hypertension (n, %)	90 (69.2)	106 (80.3)	103 (77.4)	0.096
Diabetes mellitus (n, %)	50 (38.5)	60 (45.5)	62 (46.6)	0.355
Hyperlipidemia (n, %)	74 (56.9)	89 (67.4)	78 (58.7)	0.173
Current smoker (n, %)	38 (29.2)	52 (39.4)	50 (37.6)	0.186
Body mass index (kg/m^2^)	25.7±4.7	25.6±4.0	24.9±4.1	0.267
Waist-to-hip ratio	0.92±0.08	0.94±0.07	0.94±0.08	0.038
Systolic BP (mmHg)	132±20	133±23	133±23	0.877
Diastolic BP (mmHg)	76±12	77±11	75±13	0.565
LVEF (%)	60.7±14.3	59.9±12.6	60.0±15.3	0.938
**Medications (n, %)**				
ACEIs/ARBs	9 (6.9)	13 (9.9)	11 (8.3)	0.693
CCBs	38 (29.2)	53 (40.2)	51 (38.4)	0.143
Diuretics	35 (26.9)	40 (30.3)	39 (29.3)	0.825
Thiazolidinediones	6 (4.6)	5 (3.8)	6 (4.5)	0.937
Insulin	3 (2.3)	4 (3.0)	1 (0.8)	0.404
Statins use	68 (52.3)	72 (54.6)	57 (42.9)	0.142
**Number of diseased vessels (%)**				
Single-vessel disease	33 (25.4)	19 (14.4)	18 (13.5)	0.020
Two-vessel disease	33 (25.4)	24 (18.2)	32 (24.1)	0.330
Three-vessel disease	45 (34.6)	61 (46.2)	53 (39.9)	0.159
Fatty liver (%)				
Yes	41 (31.1)	46 (35.4)	63 (47.4)	0.018
No	91 (68.9)	84 (64.6)	70 (52.6)	

Data are expressed as the mean ± SD, or number (percentage). BP, blood pressure; LVEF, left ventricular ejection fraction; ACEI, angiotensin- converting enzyme inhibitor; ARB, angiotensin receptor blocker; CCB, calcium channel blocker. Fatty liver was defined by ultrasonography and fibrosis-4 index.

**Table 2:** Biochemical characteristics according to tertiles of hepassocin

Parameter	Low hepassocin ≤ 570 ng/mL	Medium hepassocin = 571-1230 ng/mL	High hepassocin >1230 ng/mL	*p*-value
No.	130	132	133	
Fasting glucose (mg/dl)	133.5±68.4	136.4±61.8	137.1±60.3	0.888
HbA1C (%)	6.7±1.4	6.9±1.4	7.0±1.6	0.233
T-cholesterol (mg/dl)	169.1±37.8	180.5±46.5	167.6±42.6	0.028
Triglyceride (mg/dl)	115.0 (83.0-173.5)	118.0 (92.0-180.0)	109.0 (78.3-164.3)	0.261
HDL-cholesterol (mg/dl)	40.6±12.3	39.4±11.0	39.2±11.7	0.558
LDL-cholesterol (mg/dl)	100.0±35.6	106.7±36.0	98.5±35.3	0.143
Uric acid (mg/dl)	6.2±2.0	6.8±1.9	7.1±2.7	0.006
BUN (mg/dl)	17.3 (13.9-22.2)	19.6 (15.9-26.6)	20.0 (14.1-29.4)	0.027
Creatinine (mg/dl)	1.2 (1.0-1.4)	1.3 (1.1-1.7)	1.3 (1.1-1.8)	0.053
eGFR (ml/min/1.73m^2^)	63.2±22.6	53.8±22.0	51.9±24.3	0.0001
Hemoglobin (g/dL)	13.1±1.9	13.1±1.9	12.6±2.1	0.049
Albumin (g/dL)	4.0±0.4	3.9±0.4	3.8±0.4	0.036
Total WBC count (10^9^/L)	7.059±2.399	7.402±2.469	7.759±3.231	0.065
Neutrophil count (10^9^/L)	4441±2147	4569±2162	5150±2929	0.044
Monocyte count (10^9^/L)	397±189	427±182	574±303	0.041
Lymphocyte count (10^9^/L)	2004±893	2069±970	1894±715	0.253
hs-CRP (mg/L)	1.8 (0.7-5.8)	2.8 (1.0-8.9)	3.3 (1.2-10.1)	0.008
Fibrosis-4 index	1.1 (0.9-1.4)	1.8 (1.4-2.4)	3.2 (2.3-4.5)	0.0001

Data are expressed as the mean ± SD, or median (interquartile range). HDL, high-density lipoprotein; LDL, low-density lipoprotein; BUN, blood urine nitrogen; eGFR, estimated glomerular filtration rate; WBC, white blood cell; hs-CRP, high sensitivity C-reactive protein.

**Table 3:** Linear regression analysis of variables associated with plasma hepassocin levels

Variable	Simple		Multiple	
	β coefficient	*p*-value	β coefficient	*p*-value
Body mass index	-0.032	0.520	-	-
Systolic BP	0.057	0.264	-	-
Diastolic BP	-0.005	0.926	-	-
Fasting sugar	0.022	0.666	-	-
HbA1C	0.042	0.425	-	-
T-cholesterol	-0.024	0.639	-	-
Triglyceride	0.099	0.050	-	-
HDL-cholesterol	-0.033	0.516	-	-
LDL-cholesterol	-0.053	0.293	-	-
Uric acid	0.137	0.010	-	-
Blood urea nitrogen	0.139	0.006	-	-
Creatinine	0.103	0.040	-	-
eGFR	-0.183	<0.0001	-0.228	<0.0001
Hemoglobin	-0.092	0.067	-	-
Albumin	-0.022	0.675	-	-
Total WBC count	0.111	0.028	-	-
Neutrophil count	0.106	0.036	-	-
Monocyte count	0.044	0.384	-	-
Lymphocyte count	-0.020	0.699	-	-
Hs-CRP	0.108	0.041	-	-
Fibrosis-4 index	0.122	0.012	-	-

In multiple linear stepwise regression analysis, all values were included for analysis. BP, blood pressure; HDL, high-density lipoprotein; LDL, low-density lipoprotein; eGFR, estimated glomerular filtration rate; WBC, white blood cell; Hs-CRP, high sensitivity C-reactive protein.
